# International Conference on Birth Control

**Published:** 1922-08

**Authors:** 


### INTERNATIONAL CONFERENCE ON BIRTH CONTROL.

THE fifth International Conference on Birth Control was held in London on July 11–14. The attendance was not large, but there were delegates from all parts of the world, as well as a number of medical officers and other representatives of public bodies in this country.

The President, Dr. C. V. Drysdale, delivered an address. There had been, he said, an embargo on the entrance of science into the social sphere, and this movement is the result of an attempt of the greatest thinkers of the past and present to obtain a really scientific sociology. They met for the first time in the knowledge that the whole world was ready for the doctrine of birth control, and that East and West were ready to meet on this fundamental human need. The terrible economic distress and unemployment which had followed from the destruction of capital by the war had forced statesmen to recognise the fundamental truth of the Malthusian doctrine, while the great pronouncement by Lord Dawson, the King's physician, in favour of a rational and humane sexual philosophy and of birth control had had an effect over the whole world. To-day, all organised opposition to birth control was dead, except that of the Roman Catholic Church. The movement was as greatly concerned with the quality of the race as with its quantity, and the latter question was fast becoming of supreme importance in countries like Great Britain, America and France, where the birth-rate had now fallen to fairly manageable figures, but where family limitation had been adopted by the educated and successful sections, leaving the poor, improvident stocks to multiply without restraint in their ignorance, to the great injury of our race. The movement had always been deeply concerned with moral ideals, and had always had as one of its chief aims the promotion of sexual purity through the advocacy of general early marriage. One of the most important objects of the Conference was to urge that the public authorities of all nations should take steps to secure the provision of hygienic birth-control instruction at all hospitals and medical centres where the poor and diseased congregated.

For the whole of Europe the birth-rate had fallen from 40 in 1876 to 36·5 in 1901, which meant that, assuming the same rate of decline to have continued, there were now 3,000,000 fewer births annually in Europe alone; while America and Australasia probably swelled the reduction to about 5,000,000 annually. This meant that at least 20,000,000 people had adopted family limitation in some form or another in the 45 years since the commencement of the movement. The reduction of the number of births had caused the saving of at least the same number of lives. If the process was continued until the average birth-rate of the entire world was reduced to 17 or 18 per 1,000, they would save lives at the rate of 25,000,000 a year, remove untold suffering from millions of hapless men, women and children, make early marriage and social purity possible, eliminate the struggle for bare existence, improve the quality of the race, and reduce the economic rivalry which was the most potent cause of international friction and war.

Herr Ferch said that in Austria their opponents, who were supported by large sums of money, were mainly the war profiteers and shouters, who were dreaming, by the help of a surplus birth-rate, of a war of revenge.

Dr. Goldstein, the German representative, deplored the fact that nothing of the Conference would be published in the German papers.

A resolution was passed expressing pleasure at the achievements of the movement and its extension to the East, and calling upon the Governments of all nations to facilitate the extension of birth-control knowledge among the poor and hereditarily unfit, in the interests of human welfare, race improvement and lasting peace.

Mrs. Margaret Sanger (president of the American Birth Control League) presided at the afternoon meeting.

Mr. Edward Cecil, discussing "C3 Motherhood," said that the root cause of this evil was too much motherhood. What chance, under modern conditions, especially in towns, was there for the mother or the children when children were brought into the world at the rate of one per year? The mothers of the poor should be taught how their motherhood could be regulated in accordance with their means and physical capacity,

### Moral and Religious Aspects.

The Rev. Gordon Lang, who presided over this section, regarded birth-control as the fundamental solution of a fundamental problem. In South Wales there was an appalling amount of poverty. People were starving while they worked, some of them, after being paid, having to obtain parish relief. Yet in some of these homes where destitution was most rife they had the most rapid production of children.

Dr. B. Dunlop urged that birth-control was necessary for the elimination of poverty, and was therefore moral.

A resolution was carried declaring "that the practice of birth-control is not contrary to the dictates or spirit of Christianity, but has been advocated from motives of the deepest compassion for the poor and suffering, and as the only practicable means of securing the highest ideal of married and sexual purity."

